# Wafer-Scale Synthesis of WS_2_ Films with In Situ Controllable p-Type Doping by Atomic Layer Deposition

**DOI:** 10.34133/2021/9862483

**Published:** 2021-12-06

**Authors:** Hanjie Yang, Yang Wang, Xingli Zou, Rongxu Bai, Zecheng Wu, Sheng Han, Tao Chen, Shen Hu, Hao Zhu, Lin Chen, David W. Zhang, Jack C. Lee, Xionggang Lu, Peng Zhou, Qingqing Sun, Edward T. Yu, Deji Akinwande, Li Ji

**Affiliations:** ^1^State Key Laboratory of ASIC and System, School of Microelectronics, Fudan University, Shanghai 200433, China; ^2^State Key Laboratory of Advanced Special Steel, School of Materials Science and Engineering, Shanghai University, Shanghai 200444, China; ^3^Microelectronics Research Center, Department of Electrical and Computer Engineering, The University of Texas at Austin, Austin, 78758 Texas, USA

## Abstract

Wafer-scale synthesis of p-type TMD films is critical for its commercialization in next-generation electro/optoelectronics. In this work, wafer-scale intrinsic n-type WS_2_ films and in situ Nb-doped p-type WS_2_ films were synthesized through atomic layer deposition (ALD) on 8-inch *α*-Al_2_O_3_/Si wafers, 2-inch sapphire, and 1 cm^2^ GaN substrate pieces. The Nb doping concentration was precisely controlled by altering cycle number of Nb precursor and activated by postannealing. WS_2_ n-FETs and Nb-doped p-FETs with different Nb concentrations have been fabricated using CMOS-compatible processes. X-ray photoelectron spectroscopy, Raman spectroscopy, and Hall measurements confirmed the effective substitutional doping with Nb. The on/off ratio and electron mobility of WS_2_ n-FET are as high as 10^5^ and 6.85 cm^2^ V^−1^ s^−1^, respectively. In WS_2_ p-FET with 15-cycle Nb doping, the on/off ratio and hole mobility are 10 and 0.016 cm^2^ V^−1^ s^−1^, respectively. The p-n structure based on n- and p- type WS_2_ films was proved with a 10^4^ rectifying ratio. The realization of controllable *in situ* Nb-doped WS_2_ films paved a way for fabricating wafer-scale complementary WS_2_ FETs.

## 1. Introduction

As silicon-based CMOS technology is reaching its physical limits, two-dimensional transition metal dichalcogenides (TMDs) have been intensively investigated as potential ultrathin channel materials for future electronics. TMDs show tunable bandgap, good air-stability, and high carrier mobility and can be applied in transistors [1–4], photodetectors [5], computing technologies [6, 7], memory [8, 9], RF [10–12], and heterojunction synapse [13, 14]. However, there are still many challenges, including (1) realization of large wafer-scale deposition, (2) a controllable p-type doping method for TMD films, (3) reducing Schottky barrier-induced Fermi level pinning at the metal/TMDs contacts, and (4) high-quality high-k/TMD interface. Chemical vapor deposition (CVD) is an effective way to synthesize single-crystalline TMDs films [15–17], but wafer-scale deposition and precisely-controlled thickness of TMDs films are difficult to achieve via CVD. Because TMD films are too thin for p-type doping by ion implantation [18–21], a variety of different approaches have been pursued, including charge transfer doping by physical adsorption of molecules or salts on surface [22–25], and metal oxides (MoO_3_) [26] or metal-induced inversion (Tungsten) [27, 28] of WS_2_ through interfacial interactions. However, it has proven difficult to precisely control the doping behaviors and consequently electronic device performance.

Atomic layer deposition (ALD), a self-limiting process with precisely controlled layer thickness, is an ideal technique to synthesize wafer-scale TMD films [29–32]. Niobium (Nb) has been demonstrated as an effective p-type dopant for WS_2_ [33–35]. Halide-assisted CVD and low-pressure CVD have been utilized to insert Nb atoms into the WS_2_ lattice [20, 36], and pulsed laser deposition (PLD) can also achieve p-type WS_2_ films using premelted Nb-doped targets, but without device demonstration [18]. However, neither CVD nor PLD is capable of *in situ* and controllable doping. ALD has been demonstrated for the synthesis of wafer-scale WS_2_ films with WF_6_ as a W precursor and H_2_S as a S precursor [37, 38]. However, very few works have reported in situ controllable p-type-doped WS_2_ FETs through ALD [39]. NbS_2_ can be synthesized by utilizing NbCl_5_ and HMDST in ALD, similar to WS_2_. In addition, the lattice constants of 2H-NbS_2_ ((*a*, *b*, *c*) = (0.332, 0.332, 1.194) nm) are close to those of 2H-WS_2_ ((*a*, *b*, *c*) = (0.316, 0.316, 1.247) nm), which facilitates substitutional doping of Nb atoms into the WS_2_ lattice [40].

Here, in this work, we demonstrate for the first time the wafer-scale synthesis of WS_2_ films by ALD with controllable *in situ* p-type doing, on 8-inch *α*-Al_2_O_3_/Si wafer, 2-inch sapphire wafers, and 1 cm^2^ GaN substrate pieces. The growth mechanisms of ALD WS_2_ and *in situ* Nb doping were analyzed, and the doping concentration is shown to be controllable by altering Nb cycle numbers. Plan-view and cross-sectional TEM imaging reveals the layered structure of WS_2_, and Hall effect measurements and TOF-SIMS confirm the effective incorporation of Nb dopants. Moreover, both WS_2_ n-FETs and Nb-doped WS_2_ p-FETs were fabricated by CMOS-compatible processes from as-prepared ALD-grown n-WS_2_ and Nb-doped p-WS_2_ films. The on/off ratio and electron mobility of WS_2_ n-FET were up to 10^5^ and 6.85 cm^2^ V^−1^ s^−1^, while the on/off ratio and hole mobility of Nb-doped WS_2_ p-FET were 10^1^ and 0.016 cm^2^ V^−1^ s^−1^, respectively. WS_2_ FETs with different concentrations of Nb dopants were also investigated. Our work, by demonstrating *in situ* controllable Nb-doped WS_2_ films and consequently p-FETs, helps establish a path to fabricate complementary WS_2_ FETs at wafer-scale volumes.

## 2. Results

### 2.1. Growth Mechanisms

**Figure 1:**
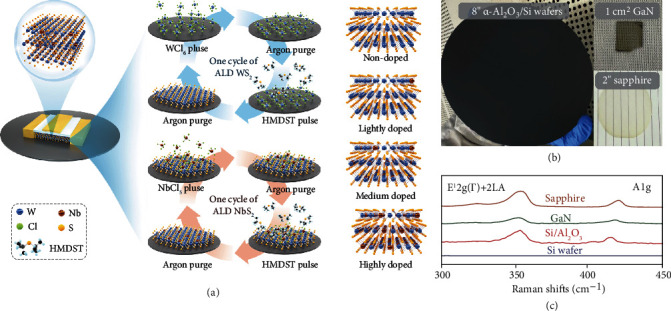
Illustration of ALD growth mechanisms and characterizations. (a) Idealized schematic of the mechanisms of ALD process for WS_2_ growth and in situ Nb doping. The doping concentration could be controlled by adjusting NbS_2_ cycle numbers. (b) Photographs of 400-cycle WS_2_ films deposited on 8-inch *α*-Al_2_O_3_/Si wafer, 2-inch sapphire wafer, and pieced GaN substrates. (c) The Raman spectra of annealed WS_2_ on Si/Al_2_O_3_, GaN, and sapphire confirm the successful synthesis of WS_2_ on each substrate surface.

Figure 1(a) illustrates the mechanisms of the ALD process for WS_2_ growth and *in situ* Nb doping. The reactor temperature was 400°C, while the WCl_6_ (99.9%), NbCl_5_, and HMDST (98%) were kept at 93°C, 60°C, and room temperature, respectively. One cycle of WS_2_ deposition includes 1 s WCl_6_ pulse, followed by 8 s purge (Argon, 99.99%), and 1 s HMDST pulse, followed by 5 s purge, sequentially. For Nb doping, NbCl_5_ and HMDST are used as precursors. One cycle of NbS_2_ deposition includes 1 s NbCl_5_ pulse, followed by 8 s purge (Argon, 99.99%), and 1 s HMDST pulse, followed by 5 s purge. The growth rate of WS_2_ film was calibrated to about 0.036 nm/cycle. To realize a controllable in situ doping, WCl_6_ pulses were replaced by NbCl_5_ pulses, and the doping concentration could thus be adjusted by varying NbCl_5_ pulse numbers. Figure 1(b) shows photographs of wafer-scale 400-cycle WS_2_ films deposited on 8-inch amorphous-Al_2_O_3_/Si wafer, 2-inch sapphire wafer, and pieced GaN substrate with good uniformity. Raman spectra of 400-cycle annealed WS_2_ films at 950°C are shown in Figure 1(c), confirming that high-quality WS_2_ could be deposited on all these substrates except for Si with different thickness at 400 cycles. In view of this, we use sapphire as the substrate for this research.

#### 2.1.1. ALD-Deposited WS_2_ Film

**Figure 2:**
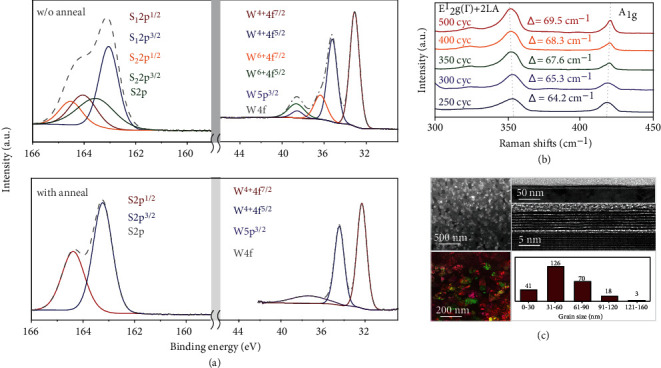
Material characterizations of ALD grown WS_2_ films without doping. (a) The XPS fine spectra of W 4f and S 2p for as-deposited and annealed 400-cycle WS_2_ film. Both WS_2_ and WS_2+x_ peaks were observed, with the W/S ratio of 1 : 2.7. Only WS_2_ was observed in fine spectra of annealed WS_2_, indicating the necessity of annealing, and the W/S ratio was reduced to 1 : 2.1. (b) The Raman spectra of annealed WS_2_ films with varying thickness. The Raman peak separation increased with increasing film thickness. (c) The plane-view and cross-sectional TEM result of 400-cycle annealed WS_2_ film and the plane-view TEM result and statistical analysis of film grains by selected area electron diffraction (SAED) patterns. A layered structure was clearly observed in cross-sectional TEM. The average grain size of 259 WS_2_ grains was 55 nm, with the maximum size up to 160 nm.

At the initial stage, the WCl_6_ and HMDST vapor were exposed directly onto the sapphire substrates and WS_2_ layers were formed laterally on sapphire substrates. The subsequent layers were deposited onto the initial WS_2_ layer to connect the isolated flakes and form films. Considering this, a postannealing process would be beneficial for improving film quality. The as-deposited WS_2_ films were annealed at 950°C for 2 h in sulfur atmosphere. The XPS spectra of as-deposited and annealed WS_2_ films are shown in Figure 2(a). The fine spectra of as-deposited WS_2_ contained two pairs of W 4f peaks, representing WS_3_ and WS_2_, respectively. The higher coordination number of W atom in WS_3_ than that in WS_2_ results a shift towards higher binding energy, with the binding energies of W^6+4^f^5/2^ and W^6+4^f^7/2^ being 38.7 eV and 36.68 eV and those of W^4+4^f^5/2^ and W^4+4^f^7/2^ being 35.22 eV and 33.08 eV, respectively. Similarly, the fine spectra of as-deposited WS_2_ showed two pairs of S 2p peaks. The positions of the S_2_ 2p^1/2^ and S_2_ 2p^3/2^ peaks for W^6+^-S bonding were at 164.54 eV and 163.54 eV, while the positions of the S_1_ 2p^1/2^ and S_1_ 2p^3/2^ peaks for W^4+^-S bonding were at 164.02 eV and 163.04 eV, respectively. XPS analysis for as-deposited WS_2_ films shows the films to be a mixture of WS_2_ and WS_3_, and the stoichiometric ratio of W/S was about 1 : 2.7. A postannealing process in S atmosphere at 950°C for 2 hours improves film crystallinity. After annealing, the fine spectra of W 4f exhibited only one pair of W 4f^5/2^ and W 4f^7/2^ peaks, indicating WS_3_ components decomposed to WS_2_, along with a similar result for S 2p spectra, both without characteristic peaks indicative of W^6+^-S bonding. As a result, the stoichiometric ratio of W/S was reduced to 1 : 2.1, with the help of desulfurization and improved film crystallinity. The full spectra of as-deposited and annealed WS_2_ are shown in Fig. S2. To further investigate the crystallinity of as-deposited and annealed WS_2_ films, Raman spectroscopy was performed. After annealing, the relative intensity of the A_1g_ and E^1^_2g_+2LA(M) peaks for annealed WS_2_ was much higher than that of as-deposited WS_2_ (Fig. S3), confirming the improved film crystallinity after annealing. Therefore, subsequent WS_2_ films in this paper have undergone a postannealing process. In addition, when increasing WS_2_ film thickness from 250 cycle to 500 cycle, the separation between the A1g and E^1^_2g_+2LA(M) peaks increased from 64.2 cm^−1^ to 69.5 cm^−1^, demonstrating good thickness controllability for ALD grown WS_2_, as shown in Figure 2(b). Plan-view and cross-sectional TEM imaging shown in Figure 2(c) reveal a continuous planar film, without warpages or kink formation. The thickness of the annealed 400-cycle WS_2_ film was 4.6 nm, and a cross-sectional TEM image of a 3.7 nm WS_2_ film is shown in Fig. S4. Preparing monolayer films is very challenging due to the growth mechanism of ALD TMD films. From the plane-view TEM and SAED patterns results, out of 259 WS_2_ analyzed grains, the average grain size was 55 nm (details of grain size were shown in Fig. S5), while the largest grain size was as high as 160 nm. The AFM image of 4.6 nm WS_2_ film is shown in Fig. S6.

#### 2.1.2. In Situ Niobium-Doped p-Type WS_2_ Films

**Figure 3:**
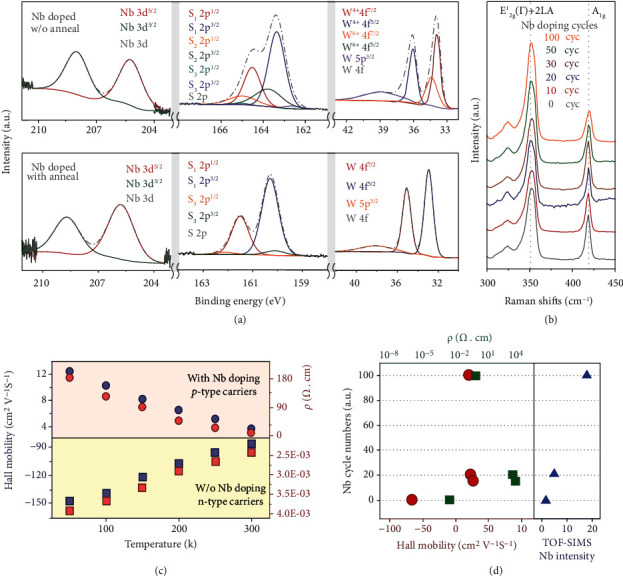
Material characterization of Nb-doped p-type WS_2_. (a) The XPS fine spectra of as-deposited and annealed 400-cycle WS_2_ with 30-cycle Nb doping. WS_2_, WS_2+x_, and NbS_2_ were all observed in as-deposited Nb-doped WS_2_ film. After annealing, only WS_2_ and NbS_2_ were observed, indicating the effective doping. (b) The Raman spectra of 400-cycle WS_2_ with Nb doping varying from 10 to 100 cycles. A blue shift of A_1g_ peak was observed when increasing doping concentration, implying the stiffening of Nb-doped WS_2_ lattice with Nb-S bonds. (c) The hall mobility and resistivity of WS_2_ and Nb-doped WS_2_ with 50-cycle Nb doping at temperature varying from 50 K to 300 K. (d) The mobility, resistivity, and TOF-SIMS of WS_2_ and Nb-doped WS_2_ with Nb doping of 15, 20, and 100 cycles. After 15-cycle Nb doping, the carrier type changed from electrons to holes, and the mobility decreased one order of magnitude, while the resistivity increased 4 orders of magnitude. However, with increasing Nb doping, the mobility continued to decrease, while the resistivity started to decrease. The normalized Nb secondary ion intensity of Nb-doped WS_2_ films indicated the occurrence of p-type doping.

Pure NbS_2_ films were deposited by ALD using NbCl_5_ and HMDST precursors, and the XPS results of as-deposited NbS_2_ films are shown in Fig. S7. The Nb doping process is illustrated in Fig. S8 and Table S1. as-deposited and annealed 400-cycle WS_2_ films with 30-cycle Nb doping were then investigated by XPS. In the fine spectra of W 4f peaks (Figure 3(a)) of as-deposited Nb-doped WS_2_ films, two pairs of characteristic peaks revealing both W^6+^-S bonding and W^4+^-S bonding were observed. However, different from the fine spectra of S 2p of as-deposited WS_2_, a pair of characteristic peaks of Nb-S bonding was also observed, indicating successful Nb substitutional incorporation. The fine spectra of Nb 3d confirmed the presence of NbS_2_ as well. After annealing, only W^4+^-S bonding was observed in the W 4f fine spectra (see Figure 3(a)), while W^4+^-S bonding and Nb-S bonding were both observed in the S 2p fine spectra. The Nb 3d fine spectra proved the formation of NbS_2_, indicating that Nb atoms were substituted into WS_2_ lattice. The stoichiometric ratio of Nb/S was about 1 : 2.0, while that of W/S was 1 : 2.1. The full spectra of as-deposited and annealed Nb-doped WS_2_ are shown in Fig. S9. The Raman spectra of annealed Nb-doped 400-cycle WS_2_ films with Nb doping varying from 10 cycles to 100 cycles are shown in Figure 3(b). From the spectra, the blue shift of the A_1g_ peaks was obvious, especially in the Nb-doped WS_2_ film with 100-cycle Nb doping, which implies stiffening of the Nb-doped WS_2_ lattice with Nb-S bonds [18]. The annealing process was necessary for Nb atoms to be activated and incorporated substitutionally into the WS_2_ lattice. The plan-view EDX mapping results are shown in Fig. S10, confirming successful Nb doping of the WS_2_ film.

Hall effect measurements of undoped WS_2_ and Nb-doped WS_2_ with 30-cycle Nb doping were performed at temperatures ranging from 50 K to 300 K. As shown in Figure 3(c), the carrier type of undoped WS_2_ was electrons, while the carrier type of Nb-doped WS_2_ film was holes, confirming the effective Nb-substitutional doping. The hall mobility of undoped WS_2_ was up to 147.9 cm^2^ V^−1^ s^−1^ at 50 K and 86.3 cm^2^ V^−1^ s^−1^ at 300 K, while the hall mobility of Nb-doped WS_2_ was 12.4 cm^2^ V^−1^ s^−1^ at 50 K and 3.6 cm^2^ V^−1^ s^−1^ at 300 K, respectively. The resistivity of Nb-doped WS_2_ was 4 orders of magnitude higher than that of WS_2_, which revealed the fact that the Nb atom was effectively doped to substitute W atom in WS_2_ lattice.

As shown in Figure 3(d), the Hall mobility and resistivity of Nb-doped WS_2_ films with Nb doping of 15, 20, and 100 cycles at 300 K and TOF-SIMS of pristine WS_2_ and Nb-doped WS_2_ with Nb doping of 20 and 100 cycles were investigated as well. With increasing Nb concentration, the hall mobility decreased from 12.60 cm^2^ V^−1^ s^−1^ to 5.73 cm^2^ V^−1^ s^−1^, while the resistivity of 15-cycle Nb-doped WS_2_ film was 3 orders of magnitude higher than that of 100-cycle Nb-doped WS_2_ film. This result implied that 100-cycle Nb-doped WS_2_ was heavily p-doped. Nb secondary ion intensity of pristine WS_2_ film was normalized to 1, while the Nb intensity of Nb-doped WS_2_ films with Nb doping of 20 and 100 cycles was normalized as 5.13 and 19.25. The increased normalized Nb intensity implied the rising doping concentration with the increase of Nb cycle number. Both Hall effect results and TOF-SIMS gave evidence of *in situ* controllable and substitutional Nb doping. An accurate quantitative value of concentration of Nb doping could not be obtained due to the poor detection accuracy and low atom collection efficiency. STEM is not applicable for ALD grown Nb-doped WS_2_ films, due to the nature of polycrystalline films yielding only the statistical results within few layers. Raw data of Hall measurements of WS_2_ and Nb-doped WS_2_ with in Figure 3(d) are shown in Table S2.

#### 2.1.3. Electrical Properties of WS_2_ n-FET and Nb-Doped WS_2_ p-FET

**Figure 4:**
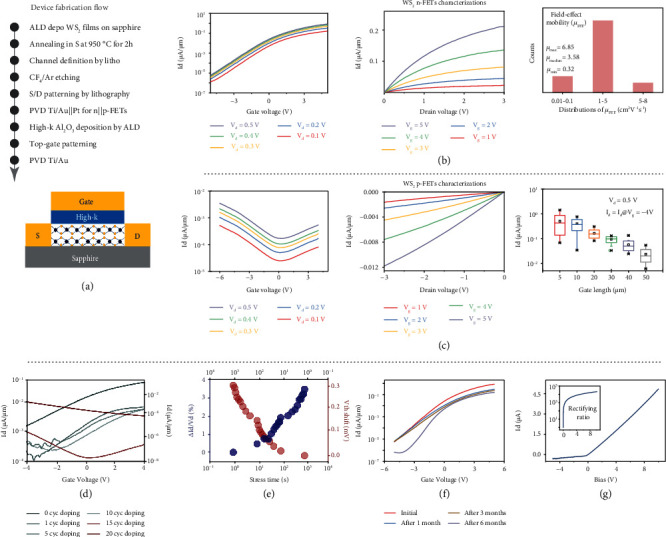
The electrical properties of WS_2_ n-FETs and Nb-doped WS_2_ p-FETs. (a) CMOS-compatible process flow of FETs and schematic of device structures. (b) The transfer and output characteristics of WS_2_ n-FET with 2 *μ*m gate width and the mobility distribution of 30 WS_2_ n-FETs. The on-current reached 0.4 *μ*A/*μ*m, and the on/off ratio was up to 10^5^. (c) The transfer and output characteristics of 15-cycle Nb-doped WS_2_ p-FET with 2 *μ*m gate width and the distribution of *I*_*d*_ at *V*_*d*_ = 0.5 V and *V*_*g*_ = −4 V for 132 Nb-doped WS_2_ p-FETs with 25-cycle Nb doping. The carrier type changed from electron to hole, and the on-current was 5 × 10^−2^ *μ*A/*μ*m. (d) The doping effects on WS_2_ FETs. Nb dopants varied from 1 to 20 cycles. Nb-doped WS_2_ FET did not show p-type behavior but with a decreased on- and off-current until reaching 15 cycles. After 20-cycle Nb doping, the device presented heavily p-type behavior, indicating the controllable doping. (e) The PBTI of WS_2_ n-FET at RT. The stress was set to be 5.5 V. After 1000 s stress, the on-current degraded only for 3.5%, while the Δ*V*_th_ was up to 300 mV. (f) The air stability of WS_2_ n-FET in ambient for 1, 3, and 6 months. The on-current degraded slightly within one order, while the degradation of off-current was less obvious after 3 months than that of 6 months. (g) The I-V curve of WS_2_/Nb-doped WS_2_ p-n structures with the rectifying ratio of over 10^4^. The inset figure was rectifying ratio.

To characterize the electrical properties of 4.6 nm WS_2_ n-FETs and Nb-doped WS_2_ p-FETs, top-gate transistors were fabricated with 2 *μ*m gate width on sapphire substrate. The CMOS-compatible process flow and the structure of top-gate FET are shown in Figure 4(a) (detailed process was discussed in Materials and Methods). ALD Al_2_O_3_ films (20 nm) were used as high-k dielectrics. The equivalent oxide thickness was 13 nm. The transfer characteristic of 8-layer WS_2_ n-FET is shown in Figure 4(b), with *V*_*d*_ varying from 0.1 V to 0.5 V, while the output characteristics with *V*_*g*_ vary from 1 V to 5 V. The transfer on-current of WS_2_ n-FET reached as high as 0.4 *μ*A/*μ*m at *V*_*d*_ = 0.5 V, and the on-off ratio was up to 10^5^. The detailed mobility of 30 tested WS_2_ n-FETs is also plotted in Figure 4(b). The maximum and minimum mobilities of n-FETs were 6.85 cm^2^ V^−1^ s^−1^ and 0.32 cm^2^ V^−1^ s^−1^, respectively, while the median mobility was 3.58 cm^2^ V^−1^ s^−1^. The mobility of over 70% of WS_2_ n-FETs was in the range of 1 to 5 cm^2^ V^−1^ s^−1^.

The transfer characteristic of a 4.6 nm Nb-doped WS_2_ p-FET with 15-cycle Nb doping with *V*_*d*_ varying from 0.1 V to 0.5 V and the output characteristics with *V*_*g*_ varying from -2 V to -6 V are shown in Figure 4(c). Compared to the WS_2_ n-FET, the carrier type changed from electron to hole, which proved the Nb substituted for W atom in WS_2_ lattice. The on- and off-current of Nb-doped WS_2_ p-FET was only 5 × 10^−2^ at *V*_*d*_ = 0.5 V, far less than that of WS_2_ n-FET. However, the hole mobility of Nb-doped WS_2_ p-FET was 0.016 cm^2^ V^−1^ s^−1^, while the on/off ratio was 10^1^. For Hall effect measurements, the resistivity of 15-cycle Nb-doped WS_2_ was 5 orders of magnitude higher than that of undoped WS_2_, and the mobility of 15-cycle Nb-doped WS_2_ was far less than that of undoped WS_2_ at 300 K. The field-effect mobility of WS_2_ FETs was smaller than the Hall effect of WS_2_, due to the influence of transistors' electrical contacts on the underestimation of field-effect mobility. The Hall mobility was roughly estimated through field-effect mobility due to the nonlinear dependence of carrier concentration on gate voltage [41]. Moreover, the stability of our process was inquired through measuring the on-current of Nb-doped WS_2_ p-FET with gate length varying from 5 *μ*m to 50 *μ*m. (Figure 4(c)). The distribution of *I*_*d*,sat_ (at *V*_*g*_ = −4 V, *V*_*d*_ = 0.5 V) amongst 132 Nb-doped WS_2_ p-FET with 20-cycle Nb doping on the same day was summarized. With increasing gate length, *I*_*d*,sat_ decreased, suggesting the fabrication process was well-controlled and uniform. To explore the controllability of Nb doping, the transfer characteristics of Nb-doped WS_2_ FETs with Nb doping varying from 1 cycle to 20 cycles were measured (Figure 4(d)). Nb-doped WS_2_ FET did not show p-type behavior but with a decreased on- and off-current until reaching 15 cycles. When further increasing Nb concentrations, the current of p-FET increased and the on/off ratio decreased in that the resistivity and mobility of Nb-doped WS_2_ film decreased, which was identical to the hall effect measurements. The WS_2_ FET was heavily p-doped after 20-cycle Nb doping. These results proved the good controllability of in situ Nb doping by ALD.

Due to the lack of dangling bonds at the surface of WS_2_, it was difficult to deposit very high quality high-k dielectrics. Thus, the PBTI of WS_2_ n-FET was carried out to analyze the reliability of Al_2_O_3_ high-k dielectric. The stress was applied to gate and biased at 5.5 V. DC transfer characteristics at *V*_*d*_ = 0.5 V were measured right after the removal of PBTI stress at room temperature. As shown in Figure 4(e), after 1000 s stress, the degradation of on-current was 3.5%, while the *V*_th_ shift was only 300 mV which was 6% of max-applied gate voltage. The results implied the instability of high-k films indeed affected the electrical properties of WS_2_ n-FET. Higher quality high-k dielectrics would improve the electrical property of WS_2_ n-FET [42]. To investigate the air stability of WS_2_ film, the WS_2_ n-FET was placed in ambient atmosphere, and the transfer characteristics were tested at *V*_*d*_ = 0.5 V after 1 month, 3 months, and 6 months, as shown in Figure 4(f). The on-current of WS_2_ n-FET degraded slightly, while the degradation was within one order of magnitude even after 6-month exposure in air. However, despite the fact that the deterioration of off-current was hardly observed after 3-month exposure, the deterioration of off-current was almost one order of magnitude after 6-month exposure. Consequently, the on/off ratio decayed from 10^5^ to 10^4^ after 6 months in ambient. Furthermore, vertical p-n structure based on WS_2_ and Nb-doped WS_2_ films was fabricated. The electrical property of p-n structure with rectifying ratio of 10^4^ is shown in Figure 4(g), with an ideal factor of 2.3, indicating a conspicuous recombination of electron-hole.

The benchmark of p-type WS_2_ transistors is listed in Table 1, including various deposition doping methods. The CVD method could yield the highest *I*_on_/*I*_off_ ratio by adjusting metal work function but suffers from the difficulties of large volume synthesis on 8/12-inch wafers. For ALD approach, wafer scale deposition has been studied; however, our work was the first demonstration of p-type WS_2_ films on large-scale wafers, with *in situ* controllable doping.

## 3. Discussion

For the first time, we demonstrated the wafer-scale synthesis of WS_2_ films by ALD with controllable *in situ* p-type doing, on 8-inch *α*-Al_2_O_3_/Si wafer, 2-inch sapphire wafer, and pieced GaN substrates with a postannealing process. The plane-view and cross-sectional TEM indicated the successful synthesis of WS_2_ film with the average grain size of 55 nm. The XPS spectra, Hall effect, and TOF-SIMS proved the substitutional doping of Nb. The Nb-doped WS_2_ FETs with different Nb doping concentrations were fabricated to demonstrate the controllable Nb doping. Furthermore, the p-n structure based on WS_2_ and Nb-doped WS_2_ films showed 10^4^ rectifying ratio, giving evidence to the realization of p-type WS_2_. Our work realized the controllable in situ Nb doping WS_2_ films by ALD, which obviated the difficulty of p-type WS_2_ film and paved a path to the fabrication of complementary WS_2_ FETs and further applications on logic circuits.

## 4. Materials and Methods

### 4.1. Material Synthesis and Characterization

The WS_2_ and Nb-doped WS_2_ film were deposited on 2-inch sapphire substrate by ALD (Beneq, TFS-200). Prior to the deposition, the sapphire substrate was cleaned by acetone, ethyl alcohol, diluted HF (1 : 50), and deionized water in order. For Nb doping, a typical cycle includes 1 s NbCl_5_ pulse, followed by 8 s purge (Argon, 99.99%), and 1 s HMDST pulse, followed by 5 s purge. To achieve Nb-doped WS_2_ film, the NbS_2_ process was sandwiched into a WS_2_ process accordingly. Nb concentration was precisely controlled through altering NbS_2_ cycle numbers. The cycle number of 4.6 nm WS_2_ was 400. The as-deposited samples were put in a quartz boat placed in the center of Zone I and Zone II, and 0.5 g sulfur powder was placed in Zone III carried by a quartz boat. The samples were annealed for 2 h in a 4-inch quartz tube at the base pressure less than 0.4 Pa. The temperature of Zone I and Zone II were raised to 950°C in 55 minutes, and the temperature of Zone III was raised to 350°C in 55 minutes. The morphology and structure of WS_2_ and Nb-doped WS_2_ were characterized by XPS (Augerscan-PHI5300, monochromatic Al K*α* anode at 9.97 kV and 14.7 mA as the source of X-ray radiation; pass energy was 112 eV; step was 0.1 eV, peak fitted using combined Gaussian, and Lorentzian line shapes), Raman (LabRAM, 532 nm laser wavelength, 1 mW x100_VIS), Hall effect measurements (Lakeshore 8400, van der Pauw, DC,4-probes), and HRTEM (Thermo Fisher Scientific Talos F200X; acceleration voltage was 200 kV; the sample was prepared by Thermo Fisher Scientific Helios G4 UX focus ion beam, and a protective layer of Pt was deposited on the surface of the sample by electron beam and ion beam).

### 4.2. Device Fabrication

Top-gate FETs for WS_2_ and Nb-doped WS_2_ films were fabricated through CMOS-compatible processes. After annealing in S atmosphere, photolithography was used to define channel area and was etched by CF_4_/Ar (20/10 sccm) in RIE. Source and drain were patterned by photolithography and metalized by Ti/Au (15/70 nm) for WS_2_ n-FETs and Pt (70 nm) for Nb-doped WS_2_ p-FETs by PVD (Kurt J. Lesker PVD75). A 20 nm Al_2_O_3_ gate oxide was deposited by ALD at 250°C. The precursors for Al_2_O_3_ were TMA and H_2_O, respectively. After top-gate patterning, 15/70 nm Ti/Au was deposited by PVD.

### 4.3. Device Measurement

All electrical properties of WS_2_ n-FETs and Nb-doped WS_2_ p-FETs were measured in ambient room temperature by the Agilent B1500A Semiconductor Device Analyzer in probe station (MPI-TS3000). The field-effect carrier mobility was extracted from the transfer characteristic using the equation *μ* = (Δ*I*_*d*_/Δ*V*_*g*_) × *L*/(*WC*_*ox*_*V*_*d*_), and the *C*_*ox*_ = 2.656 F/m^2^ was the unit gate capacitance between channel and top-gate (*C*_*ox*_ = *ε*_1_*ε*_o_/*d*, *ε*_1_ = 6, and *d* = 20 nm for Al_2_O_3_ dielectric).

## Figures and Tables

**Table 1:** Benchmark of p-type WS_2_ transistors.

Reference	Method of p-type doping	Growth method	Controllable doping	Wafer scale synthesis	EOT (nm)	*I*_on_/*I*_off_ at 4.6 MV/cm
Our work	Nb	ALD	√	√	13	10^1^
[20]	Nb	CVD	×	×	270	<10
[36]	Nb	CVD	×	×	285	10^2^
[39]	Φ_m_	ALD	×	√	15	10^2^
[27]	Φ_m_	ALD	×	√	60	10^4^
[28]	Φ_m_	CVD	×	×	5.16	10^6^
[23]	CH	PECVD	×	×	32.5	10^4^

*Φ*_m_ stands for adjusting metal work function (Ti et al.).

## Supplementary Materials

Supplementary Materials Fig. S1: thickness of the 400-cycle WS_2_ films as a function of HMDST and WCl_6_ precursor pulse time. Fig. S2: XPS full spectra of as-deposited and annealed WS_2_ films. Fig. S3: Raman spectra of as-deposited WS_2_ film. Fig. S4: cross-sectional TEM of 3.7 nm WS_2_ film. Fig. S5: grain size analysis of WS_2_ film. Fig. S6: WS_2_ film images with different cycle numbers and AFM image of 4.6 nm WS_2_ film. Fig. S7: XPS results of as-deposited NbS_2_ film. Table S1: WS_2_ film process cycles with different Nb doping concentrations. Fig. S8: schematic diagram of process cycle of Nb-doped WS_2_ film. Fig. S9: XPS full spectra of as-deposited and annealed Nb-doped WS_2_ films. Fig. S10: plane-view EDX mapping of Nb-doped WS_2_ film. Table. S2: hall measurements of WS_2_ and Nb-doped WS_2_ with Nb doping of 15, 20, and 100 cycles.
