# Supplementary material for: Wafer-Scale Synthesis of WS2 Films with In Situ Controllable p-Type Doping by Atomic Layer Deposition
Source: Research (Wash D C). 2021 Dec 6;2021:9862483. doi: 10.34133/2021/9862483 (PMC8672204; doi:10.34133/2021/9862483)
Supplement: Supplementary Materials — Fig. S1: thickness of the 400-cycle WS2 films as a function of HMDST and WCl6 precursor pulse time. Fig. S2: XPS full spectra of as-deposited and annealed WS2 films. Fig. S3: Raman spectra of as-deposited WS2 film. Fig. S4: cross-sectional TEM of 3.7 nm WS2 film. Fig. S5: grain size analysis of WS2 film. Fig. S6: WS2 film images with different cycle numbers and AFM image of 4.6 nm WS2 film. Fig. S7: XPS results of as-deposited NbS2 film. Table S1: WS2 film process cycles with different Nb doping concentrations. Fig. S8: schematic diagram of process cycle of Nb-doped WS2 film. Fig. S9: XPS full spectra of as-deposited and annealed Nb-doped WS2 films. Fig. S10: plane-view EDX mapping of Nb-doped WS2 film. Table. S2: hall measurements of WS2 and Nb-doped WS2 with Nb doping of 15, 20, and 100 cycles. [file 9862483.f1.docx]

Supplementary Materials for

**Wafer-scale synthesis of WS_2_ films with *in-situ* controllable p-type doping by atomic layer deposition**

Hanjie Yang, Yang Wang, Xingli Zou, Rongxu Bai, Zecheng Wu, Sheng Han, Tao Chen, Shen Hu, Hao Zhu, Lin Chen, David W. Zhang, Jack C. Lee, Xionggang Lu, Peng Zhou, Qingqing Sun*, Edward T. Yu*, Deji Akinwande*, Li Ji^*^

**This PDF file includes:**

Fig. S1 Thickness of the 400-cycle WS_2_ films as a function of HMDST and WCl_6_ precursor pulse time.

Fig. S2. XPS full spectra of as-deposited and annealed WS_2_ films

Fig. S3. Raman spectra of as-deposited WS_2_ film

Fig. S4. Cross-sectional TEM of 3.7 nm WS_2_ film

Fig. S5. Grain size analysis of WS_2_ film

Fig. S6. WS_2_ film images with different cycle numbers and AFM image of 4.6 nm WS_2_ film

Fig. S7. XPS results of as-deposited NbS_2_ film

Table. S1. WS_2_ film process cycles with different Nb doping concentrations

Fig. S8. Schematic diagram of process cycle of Nb doped WS_2_ film.

Fig. S9. XPS full spectra of as-deposited and annealed Nb-doped WS2 films

Fig. S10. Plane-view EDX mapping of Nb-doped WS2 film

Table. S1. WS2 film process cycles with different Nb doping concentrations

Table. S2. Hall measurements of WS2 and Nb-doped WS2 with Nb doping of 15, 20 and 100 cycles.

1. Materials characterization of WS_2_ film


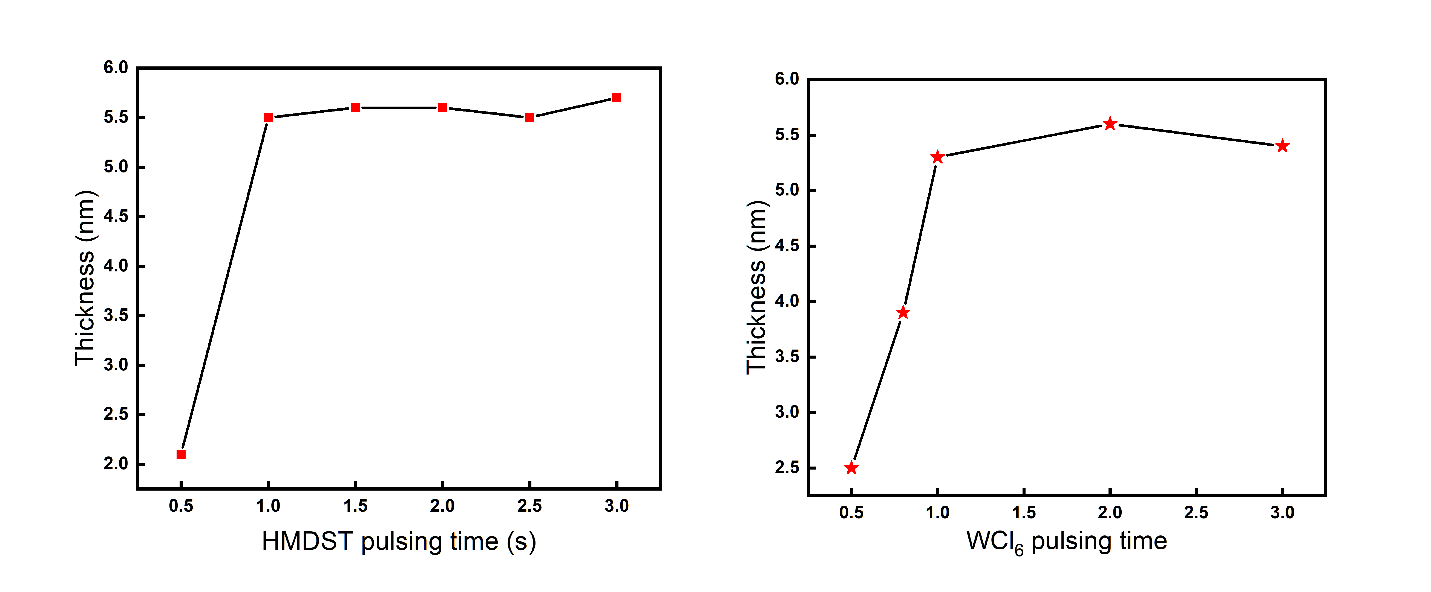


**Fig. S1 Thickness of the** **400-cycle WS_2_ films as a function of HMDST and** **WCl_6_ precursor pulse time.** The reactor temperature was 400 ℃, while the WCl_6_ (99.9 %), and HMDST (98 %) were kept at 93 ℃ and room temperature, respectively.


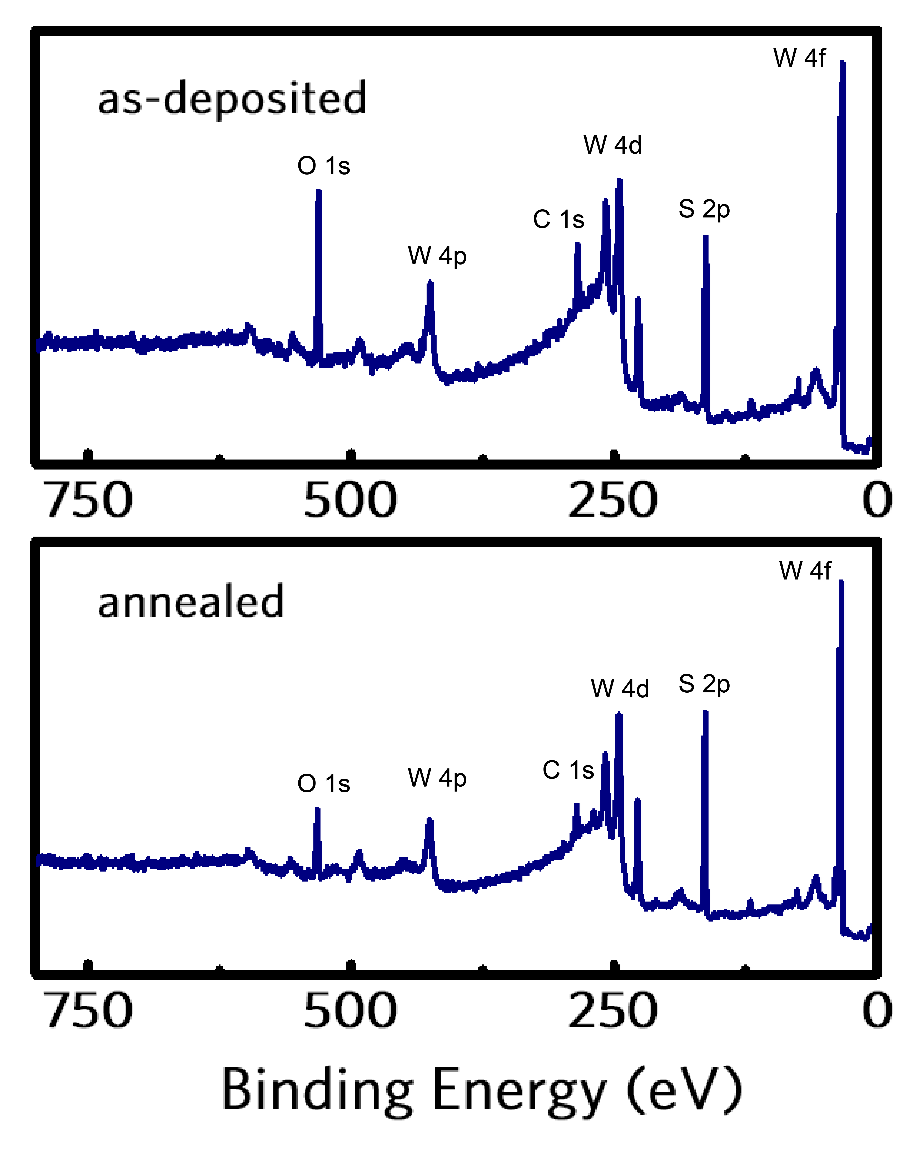


**Fig. S2. The full spectra of as-deposited and annealed WS_2_ film.** The full spectra of as-deposited and annealed WS_2_ film on sapphire was shown in Fig. S1. O 1s and C 1s peaks were observed due to the atmosphere of XPS equipment, and C 1s peaks at 284.5 eV was used for calibration. Meanwhile, S 2p, W 4f and W 4d peaks were all clearly observed in both WS_2_ full spectra, indicating the successful deposition of WS_2_ film. The annealing process took place at 950 ^o^C in S atmosphere for 2h. After annealing, the relative intensity of C and O became lower. The W/S ratio of as-deposited and annealed WS_2_ was calculated to be 1/2.7 and 1/2.1 respectively. The reduction of sulfur in annealed WS_2_ film suggested the necessity of annealing process.


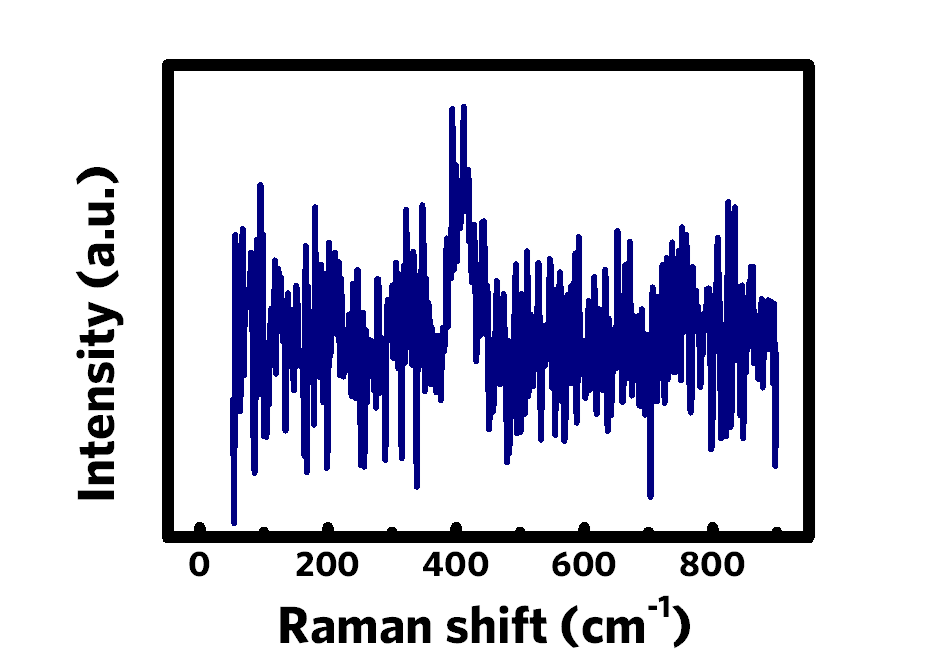


**Fig. S3. The Raman spectra of as-deposited WS_2_.** The Raman spectra of as-deposited WS_2_ film was shown in Fig. S2 with a 532 nm laser. The vibration peaks of WS_2_ were hardly observed other than the vibration peak of sapphire substrates, which implied the crystallinity was necessary to be improved. On contrast, the vibration peaks of annealed WS_2_ films were clearly observed, which proved the improvement of annealing.


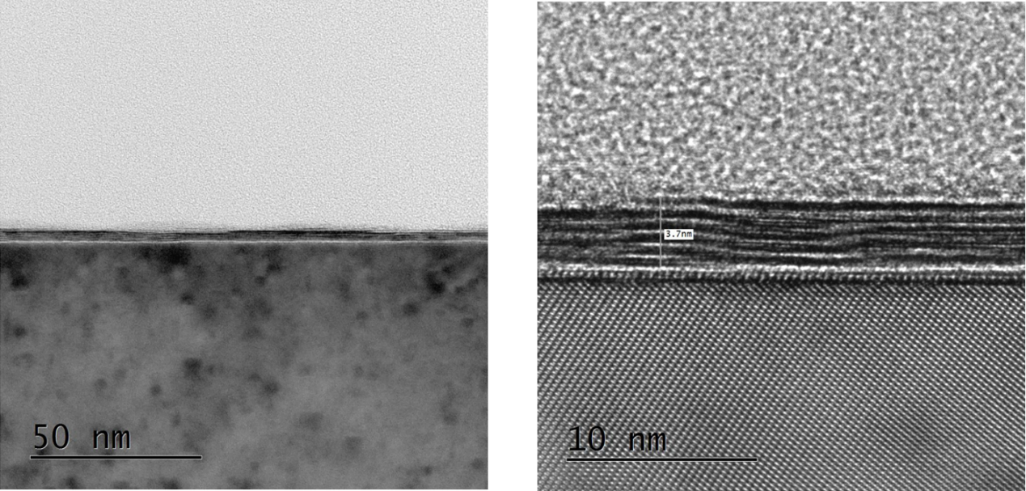


**Fig. S4.** **The cross-sectional TEM image of 3.7 nm WS_2_ film.** The cross-sectional TEM image of 3.7 nm WS_2_ film was shown in Fig. S3. Continuous planar film was observed without warpages or kinks formation. The layered-structure of WS_2_ was clearly observed but less flat than that of 4.6 nm WS_2_ film.


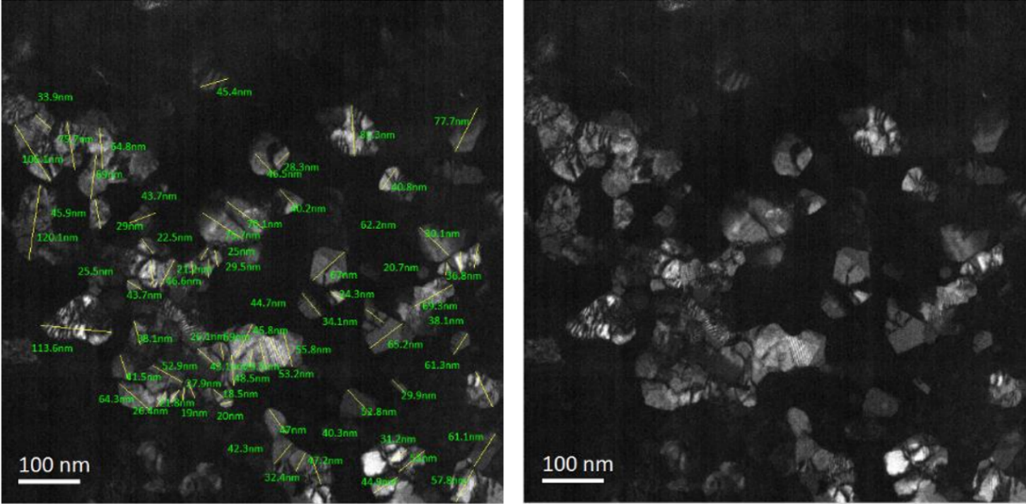


Fig. S5. The details of grain size analysis of WS_2_ grains. The detailed grain size analysis was shown in Fig. S4. Most WS_2_ grains distributed in the range of 30 to 100 nm, while some grains reached over 100 nm. Out of 259 WS_2_ grains, the average grain size was 55 nm. The grain size analysis indicated the good quality of annealed WS_2_ film.


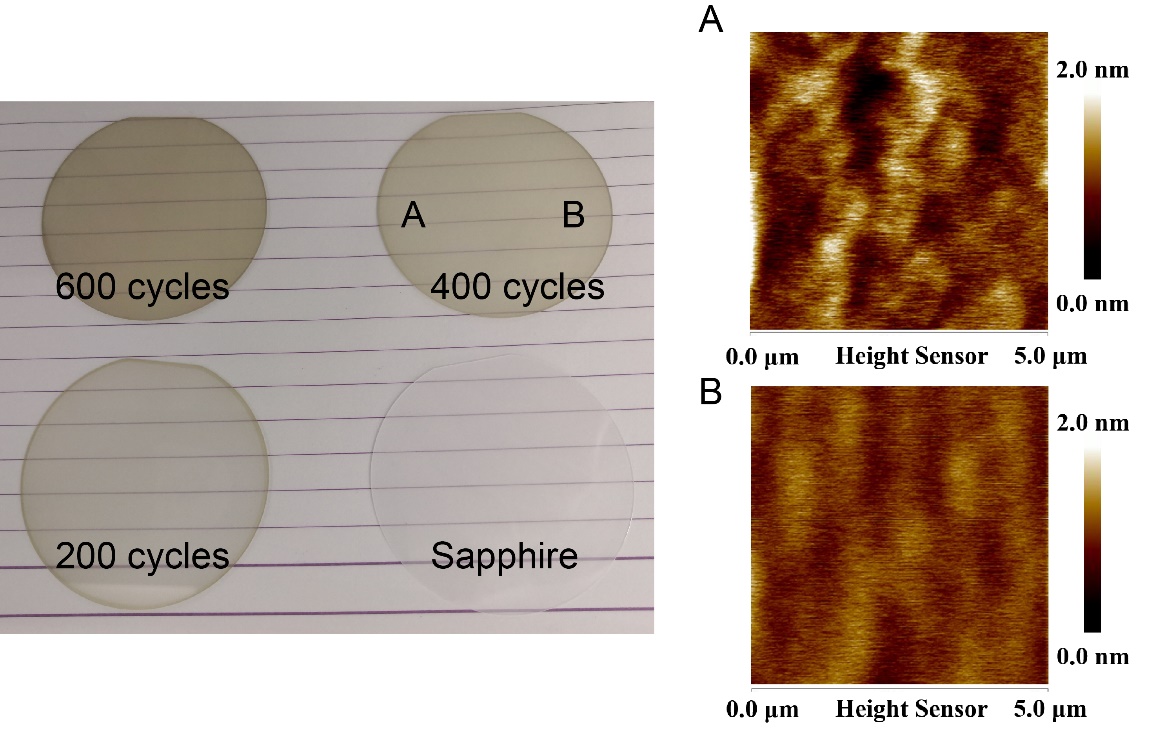


Fig. S6. WS_2_ film images with different cycle numbers prepared on 2-inch sapphire and the AFM image of 4.6 nm WS_2_ film. The root mean square (RMS) of WS_2_ film were only 0.261 nm (A) and 0.134 nm (B), indicating the good flatness of WS_2_ film. The AFM result gave evidence to the uniformity of wafer-scale WS_2_ film.

1. Materials characterization of Nb-doped WS_2_ film


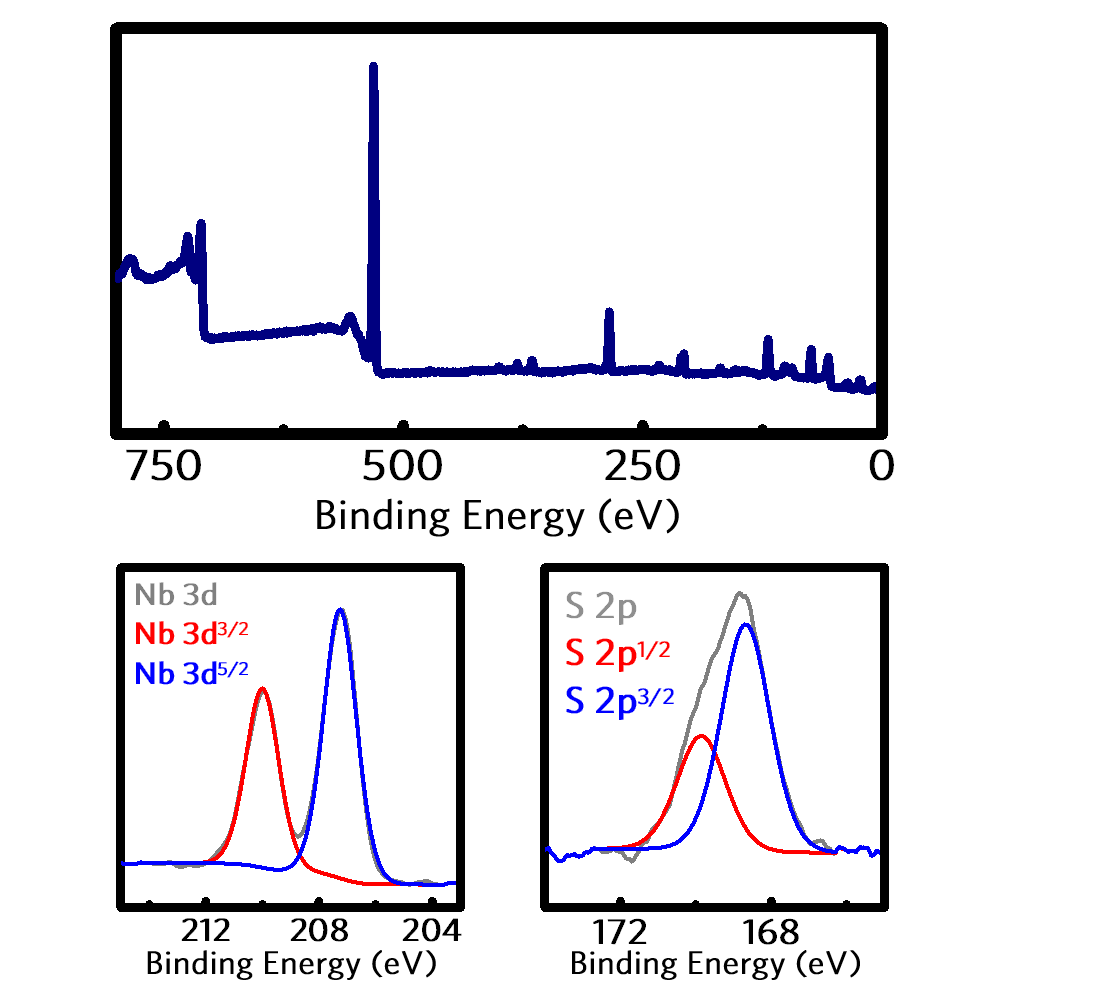


**Fig. S7.** **The XPS full spectra of as-deposited NbS_2_, and the fine spectra of Nb 3d and S 2p peaks.** The XPS results of as-deposited NbS_2_ film was shown in Fig. S6. From the full spectra of NbS_2_, the relative intensity of O 1s and C 1s peaks were more obvious than that of Nb 3d and S 2p peaks in that the thickness of NbS_2_ was very thin. The growth rate of NbS_2_ was altered to be very slow so that the doping concentration could be precisely controlled. Clearly, the observation of S 2p^1/2^, S 2p^3/2^, Nb 3d^3/2^ and Nb 3d^5/2^ peaks suggested the formation of Nb-S bonding, while the Nb/S ratio was calculated to be 1/2.0. The XPS results implied the successful synthesis of NbS_2_ film, which paved a way to *in-situ* Nb-doped WS_2_ films.

**Table. S1. WS_2_ film process cycles with different Nb doping concentrations**

| WS**_2_**  **cycles** | m | n | *l* | WS**_2_**  **cycles** | NbS**_2_**  **cycles** |
| --- | --- | --- | --- | --- | --- |
| 200 | 200 | 1 | 1 | 400 | 1 |
| 200 | 40 | 1 | 5 | 400 | 5 |
| 200 | 20 | 1 | 10 | 400 | 10 |
| 200 | 40 | 3 | 5 | 400 | 15 |
| 200 | 20 | 2 | 10 | 400 | 20 |
| 200 | 10 | 3 | 20 | 400 | 30 |
| 200 | 20 | 5 | 10 | 400 | 50 |
| 200 | 10 | 5 | 20 | 400 | 100 |


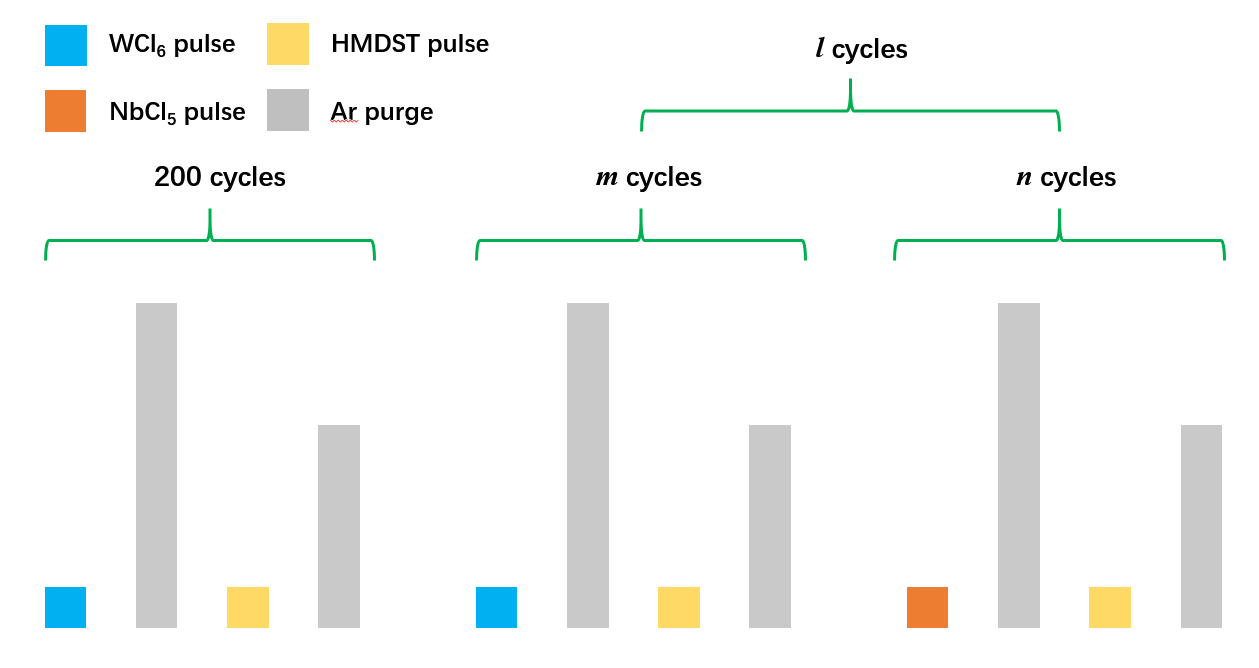


**Fig. S****8. Schematic diagram of process cycle of Nb doped WS_2_ film.** The first 200 cycles are within nucleation stage. Considering this, 200 cycles of WS_2_ pulses are performed before introducing doping cycle. The Nb doping process consists of *l* large cycles, and a large cycle contains *m* WS_2_ cycles and *n* NbS_2_ cycles.


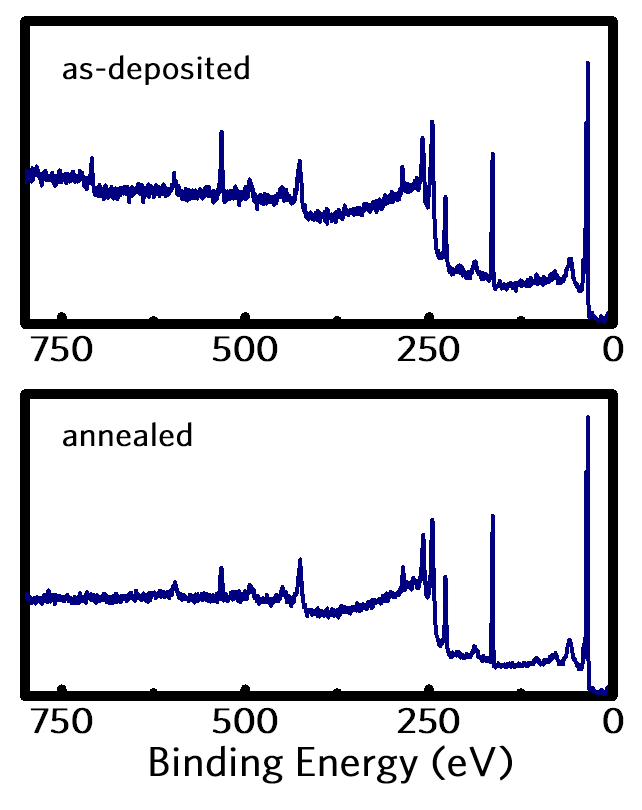


**Fig. S9.** **The full spectra of as-deposited and annealed Nb-doped WS_2_ films with 30 cycles Nb doping.** The full spectra of as-deposited and annealed Nb-doped WS_2_ film with 30 cycles Nb doping on sapphire was shown in Fig. S7. S 2p, Nb 3d, W 4f and W 4d peaks were all clearly observed in both Nb-doped WS_2_ full spectra. However, the relative intensity of Nb 3d peaks became lower after annealing process, probably because of the recombination of Nb atoms in WS_2_ lattice during annealing process. The XPS results suggested the successful doping of Nb.


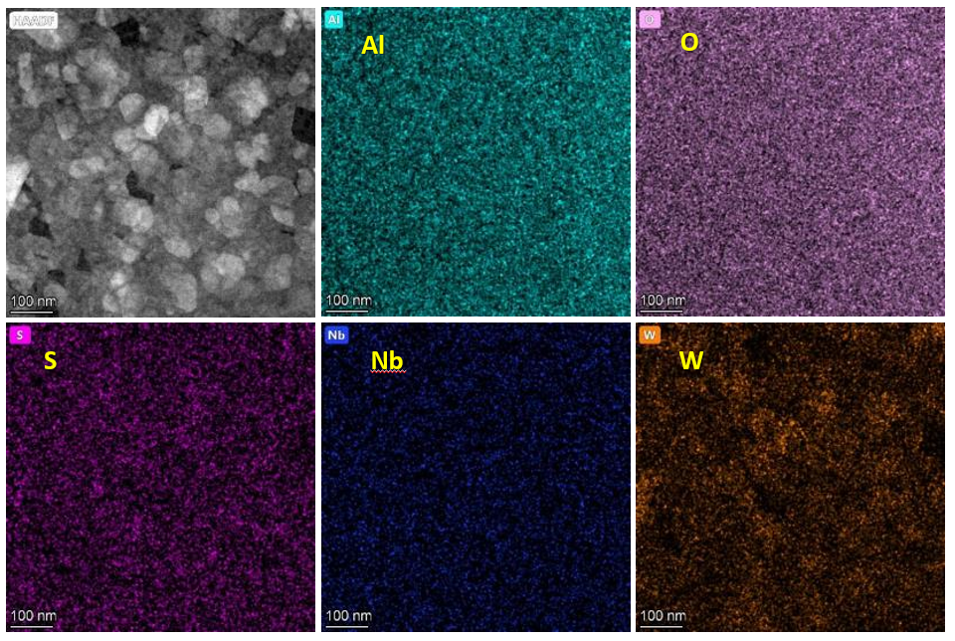


**Fig. S10.** **The plane-view EDX mapping of Nb-doped WS_2_ film.** The plane-view EDX mapping of Nb-doped WS_2_ film was shown in Fig. S8. The grains were clearly observed in HAADF TEM image, which indicated the good quality of Nb-doped WS_2_ film. The observation of Al and O elements was due to the sapphire substrates. The concentration of Nb element was far less obvious than that of S and W elements. The EDX mapping results indicated the successful doping of WS_2_ film.

**Table. S2.** **Hall measurements of WS_2_ and Nb-doped WS_2_ with Nb doping of 15, 20 and 100 cycles.**

| Temperature  (K) | Carrier type | Carrier planar density  (cm^-2^) | Carrier volume density  (cm^-3^) | Hall coefficient (cm^3^/C) | Resistivity (Ωcm) | Mobility  (cm^2^/Vs) | f factor |
| --- | --- | --- | --- | --- | --- | --- | --- |
| 0 cycle  #300 K | n | 2.09229E13 | 2.98899E19 | -0.20877 | 0.00242 | 86.33037 | 0.98594 |
| 15 cycle  #300 K | p | 6.20704E8 | 1.55176E15 | 4021.23965 | 319.0141 | 12.60217 | 0.97362 |
| 20 cycle #300 K | p | 1.56369E9 | 3.90923E15 | 1596.22043 | 194.85158 | 8.19001 | 0.98135 |
| 100 cycle #300 K | p | 2.77521E12 | 3.96459E18 | 1.57393 | 0.2745 | 5.7324 | 0.98017 |
